# Population Attributable Fraction of Smoking and Metabolic Syndrome on Cardiovascular Disease Mortality in Japan: a 15-Year Follow Up of NIPPON DATA90

**DOI:** 10.1186/1471-2458-10-306

**Published:** 2010-06-03

**Authors:** Naoyuki Takashima, Katsuyuki Miura, Atsushi Hozawa, Aya Kadota, Tomonori Okamura, Yasuyuki Nakamura, Takehito Hayakawa, Nagako Okuda, Akira Fujiyoshi, Shin-ya Nagasawa, Takashi Kadowaki, Yoshitaka Murakami, Yoshikuni Kita, Akira Okayama, Hirotsugu Ueshima

**Affiliations:** 1 Department of Health Science, Shiga University of Medical Science, Seta Tsukinowa-cho, Otsu, Shiga 520-2192, Japan; 2 Department of Public Health, Yamagata University School of Medicine, Iida Nishi 2-2-1, Yamagata, Yamagata, 990-9585, Japan; 3 Department of Preventive Cardiology, National Cardiovascular Center, Fujishirodai 5-7-1, Suita, Osaka, 565-8565, Japan; 4 The Cardiovascular Epidemiology, Kyoto Women's University, Imakumanokitahiyoshi-cho35, Kyoto, Kyoto, 605-8501, Japan; 5 Department of Hygiene and Preventive Medicine, Fukushima Medical University, Hikarigaoka1, Fukushima, Fukushima, 960-1295, Japan; 6 The First Institute for Health Promotion and Health Care, Japanese Anti-Tuberculosis Association, Misaki-cho1-3-12, Chiyoda, Tokyo, 101-061, Japan

## Abstract

**Background:**

Smoking and metabolic syndrome are known to be related to cardiovascular diseases (CVD) risk. In Asian countries, prevalence of obesity has increased and smoking rate in men is still high. We investigated the attribution of the combination of smoking and metabolic syndrome (or obesity) to excess CVD deaths in Japan.

**Methods:**

A cohort of nationwide representative Japanese samples, a total of 6650 men and women aged 30-70 at baseline without history of CVD was followed for 15 years. Multivariate-adjusted hazard ratio for CVD death according to the combination of smoking status and metabolic syndrome (or obesity) was calculated using Cox proportional hazard model. Population attributable fraction (PAF) of CVD deaths was calculated using the hazard ratios.

**Results:**

During the follow-up period, 87 men and 61 women died due to CVD. The PAF component of CVD deaths in non-obese smokers was 36.8% in men and 11.3% in women, which were higher than those in obese smokers (9.1% in men and 5.2% in women). The PAF component of CVD deaths in smokers without metabolic syndrome was 40.9% in men and 11.9% in women, which were also higher than those in smokers with metabolic syndrome (7.1% in men and 3.9% in women).

**Conclusion:**

Our results indicated that a large proportion of excess CVD deaths was observed in smokers without metabolic syndrome or obesity, especially in men. These findings suggest that intervention targeting on smokers, irrespective of the presence of metabolic syndrome, is still important for the prevention of CVD in Asian countries.

## Background

Obesity and clustering of its related factors, now called as metabolic syndrome, have been widely reported as important risk factors for cardiovascular diseases (CVD) [1-6], and, also in Asian countries including Japan, obesity has emerged as a new health problem [5]. The National Health and Nutrition Survey in Japan in 2005 showed that 22.4% of adult men and 10.8% of adult women were diagnosed as having metabolic syndrome [7]. Therefore, it is expected that metabolic syndrome or obesity would contribute to a large part of excess CVD events in Japan.

On the other hand, cigarette smoking is an established risk factor for CVD [8-12] and one of the biggest health problems in Asian countries including Japan [9,12,13]. In Asian countries, smoking rate in men is still high at 40 to 50% [14]. In Japan, smoking rate in 2005 was also high at 39.3% in men [15]. Therefore, smoking has largely contributed to increase CVD events in Asia, and it was reported that up to 30% of cardiovascular deaths was attributed by smoking in Asia Pacific region [16].

However, there have been few reports on the attribution of the combination of smoking status and metabolic syndrome (or obesity) to CVD deaths in Asian countries. Several previous studies also reported that CVD risk was high in both smokers and non-smokers with clustering of metabolic risk factors [17]. Therefore, it is important to elucidate the attribution of obesity, metabolic syndrome, and smoking to CVD mortality in Asia, where obesity is still less common compared with Western countries.

The purpose of this report is to examine excess CVD deaths and population attributable fractions on CVD deaths by the combination of smoking and metabolic syndrome (or obesity) in a 15-year cohort study of randomly selected representative Japanese samples from the National Survey on Circulatory Disorders of Japan.

## Methods

### Participants and follow-up

Cohort studies of the National Survey on Circulatory Disorders of Japan comprise the National Integrated Project for Prospective Observation of Non-communicable Disease and Its Trends in the Aged (NIPPON DATA). Baseline surveys for the cohort of this report were performed in 1990 (NIPPON DATA90) [18,19]. We analyzed the 15-year follow-up data of NIPPON DATA90 in this report.

A total of 8383 men and women aged ≥ 30 years from 300 randomly selected districts were participated in the survey in 1990. The baseline surveys were carried out at local public health centers. The participation rate in the baseline survey was 76.5%. The present study was for 7329 participants aged 30 to 70 years at baseline. From these participants, we excluded 379 participants who had a history of coronary heart disease or stroke (n = 249) or who had missing information in the baseline survey (n = 130). Thus, 6650 participants (2752 men and 3898 women) were eligible for the analyses.

NIPPON DATA90 has completed follow-up surveys until 2005. We used the National Vital Statistics data to indentify the cause of death. The underlying causes of death in the National Vital Statistics were coded according to the 9^th ^International Classification of Disease (ICD-9) until the end of 1994 and according to the 10^th ^International Classification of Disease (ICD-10) from 1995. Deaths from any CVD were identified by ICD-9 codes (393-459) and ICD-10 codes (I00-I99). The details of the classification are described elsewhere [18,19]. The Institutional Review Board of Shiga University of Medical Science (NO.12-18, 2000) approved the study.

### Biochemical and physical examinations

Public health nurses obtained data including smoking habit, as well as current health status and medical history. Public health nurses asked all participants about current smoking status (current smoking, past smoking and never-smoking), the number of cigarettes per day and the duration of smoking. Smoking habit was categorized into non-smoker, past smoker and current smoker. Drinking habit was categorized into non-drinker, past drinker, occasional drinker and daily drinker. Body mass index was calculated as weight divided by height squared (kg/m^2^).

Non-fasting blood samples were obtained at the baseline survey. The serum was separated and centrifuged soon after blood coagulation. Plasma samples were collected in siliconized tubes containing sodium fluoride and shipped to one laboratory (SRL, Tokyo, Japan) for blood measurements. Plasma glucose was measured enzymatically. Serum triglycerides and total cholesterol were also measured enzymatically, and high density lipoprotein (HDL) cholesterol was measured after heparin-calcium precipitation [20].

We defined metabolic risk factors using the Japanese criteria of the metabolic syndrome [21,22] as follow: obesity as body mass index ≥ 25 kg/m^2^; high blood pressure: BP ≥ 130/85 mm Hg or on treatment for hypertension; high blood glucose: serum glucose ≥ 110 mg/dl or on treatment for diabetes; dyslipidemia: serum triglyceride ≥ 150 mg/dl, HDL cholesterol < 40 mg/dl or on treatment for dyslipidemia. We defined the metabolic syndrome as having obesity (defined as body mass index ≥ 25 kg/m^2^) and two or more other metabolic risk factors; the definition was modified from the Japanese criteria [22] where the presence of obesity is essential. We defined metabolic risk factors clustering as having two or more metabolic risk factors.

### Statistical analysis

Multivariate-adjusted hazard ratios (HR) of all CVD deaths for each component of metabolic risk factors including BMI, systolic BP (SBP), triglyceride, glucose, HDL cholesterol were calculated using Cox proportional hazards models. Multivariate-adjusted HR for all CVD deaths according to metabolic risk factors and smoking categories were calculated using Cox proportional hazards models adjusted for age and drinking. Non-smokers without metabolic syndrome or obesity were set as the reference group.

Population attributable fractions (PAF) for CVD deaths due to the combination of smoking and metabolic syndrome (or obesity) were calculated based on hazard ratios assessed by proportional hazards models [23,24]. PAF was estimated as *pd *× (HR-1)/HR where *pd *is the proportion of death cases arising from the each categories. All analyses were performed by SAS 9.1 (Statistical Analysis System, Cary, NC).

## Results

Baseline characteristics are shown in Table 1. Mean age at baseline was 49.9 years in men and 49.0 years in women. Mean body mass index was 23.1 kg/m^2 ^in men and 22.9 kg/m^2 ^in women. Smoking rate was 58.0% in men and 9.6% in women. The prevalence of hypertension and obesity were 66.9% and 25.1% in men and 54.4% and 23.4% in women.

**Table 1:** Baseline characteristics of study population. NIPPON DATA90, men and women aged 30 to 70 years in 1990.

	Men		Women	
Number (N)	2752		3898	
Age (year)	49.9	±11.2	49.0	±11.3
BMI (kg/m^2^)	23.1	±3.0	22.9	±3.3
SBP (mmHg)	136.2	±19.5	131.3	±19.9
DBP (mmHg)	83.8	±11.7	79.4	±11.8
Total cholesterol (mg/dl)	199.6	±36.6	205.5	±38.0
HDL cholesterol (mg/dl)	50.4	±15.0	57.5	±14.9
Triglyceride (mg/dl)	151.8	±108.8	119.1	±79.8
Blood glucose (mg/dl)	102.0	±33.4	101.1	±28.9
Drinking				
Non drinker	921	33.5%	3572	91.6%
Ex-drinker	141	5.1%	39	1.0%
Current drinker	1690	61.4%	287	7.4%
Smoking				
Never smoker	556	20.2%	3431	88.0%
Ex-smoker	601	21.8%	94	2.4%
Current smoker	1595	58.0%	373	9.6%
Obesity	689	25.1%	912	23.4%
High blood pressure	1840	66.9%	2119	54.4%
High blood glucose	578	21.0%	829	21.3%
Dyslipidemia	1280	46.5%	1094	28.1%

Values are number, %, or mean ± SD.

High blood pressure, BP ≥ 130/85 mmHg or on treatment of hypertension; high blood glucose, blood glucose≥ 110 mg/dl or on treatment of diabetes; dyslipidemia as triglyceride≥ 150 mg/dl or high density lipoprotein < 40 mg/dl or on treatment of dyslipidemia.

BMI, body mass index; SBP, systolic blood pressure; DBP, diastolic blood pressure; HDL, high density lipoprotein.

During 15 years of follow-up, 87 men and 61 women died due to CVD (37 men and 22 women died due to stroke and 30 men and 8 women died due to coronary heart disease). Table 2 shows HRs of CVD death for each component of metabolic risk factors including all factors in a model, simultaneously. It showed that current smoking, past-smoking, SBP and glucose were significant risk factors of CVD mortality. Table 3 shows adjusted HRs and PAFs for CVD deaths according to the combination of obesity and smoking status. Irrespective of obesity, smoking and CVD mortality in both men and women were positively related. HRs (95% confidence interval [CI]) for non-obese smokers was 3.13 (1.33 to 7.36) in men and 4.32 (1.99 to 9.37) in women compared with non-obese, non-smokers. Estimated numbers of excess CVD deaths (and PAF component) in the non-obese smokers and obese smokers were 32.0 (36.8%), and 7.9 (9.1%) in men and 6.9 (11.3%) and 3.2 (5.2%) in women. The sum of the estimated number of excess CVD deaths (PAF) due to smoking and/or obesity was 49.3 (56.9%) in men and 15.3 (25.1%) in women.

**Table 2:** Adjusted HR for 1 standard deviation increasing in the continuous variables and sex, smoking and drinking habits for mortality from cardiovascular diseases.

	Adjusted hazard ration (95%CI)		
Current -smoker	3.45	(2.12	-5.60)
Past-smoker	2.04	(1.11	-3.75)
Body mass index (1 SD increasing)	0.99	(0.83	-1.18)
Systolic blood pressure (1 SD increasing)	1.32	(1.13	-1.54)
Triglyceride (1 SD increasing)*	0.85	(0.69	-1.04)
High density lipoprotein cholesterol (1 SD increasing)	0.93	(0.76	-1.11)
Glucose (1 SD increasing)	1.10	(1.00	-1.24)
Female	1.00	(0.61	-1.64)

This Cox model also includes age, and drinking habit.

* The variable was tested after log-transferred.

**Table 3:** Hazard ratio and population attributable fraction for cardiovascular disease deaths according to the combination of smoking status and obesity*: NIPPON DATA90

		Number of participants	Person-years of follow-up	CVD deaths (n)	CVD mortality rate (per 1,000 person-years)	Adjusted hazard ratio (95% CI)†			Estimated excess CVD deaths (n)	PAF component for CVD deaths (%)
Men										
Non smoker	Non-obese	420	5938	6	1.01	1.00				
	Obese	136	1988	1	0.50	0.67	(0.08	-5.53)	--	--
Past smoker	Non-obese	431	6116	16	2.62	1.93	(0.75	-4.96)	7.7	8.8
	Obese	170	2414	5	2.07	1.52	(0.46	-4.99)	1.7	2.0
Smoker	Non-obese	1212	16780	47	2.80	3.13	(1.33	-7.36)	32.0	36.8
	Obese	383	5277	12	2.27	2.92	(1.09	-7.82)	7.9	9.1
Women										
Non smoker	Non-obese	2,638	37960	29	0.76	1.00				
	Obese	793	11256	17	1.51	1.34	(0.74	-2.45)	4.3	7.1
Past smoker	Non-obese	66	843	1	1.19	1.43	(0.19	-10.61)	0.3	0.5
	Obese	28	383	1	2.61	2.46	(0.33	-18.09)	0.6	1.0
Smoker	Non-obese	282	3889	9	2.31	4.32	(1.99	-9.37)	6.9	11.3
	Obese	91	1224	4	3.27	4.74	(1.66	-13.58)	3.2	5.2

*Obesity was defined as body mass index ≥ 25 kg/m^2^.

†Hazard ratios were adjusted for age and drinking.

CVD, cardiovascular diseases; PAF, population attributable fraction; CI, confidence interval.

Table 4 shows adjusted HRs and PAF components due to combination of smoking status and metabolic syndrome. Compared to non-smokers without metabolic syndrome, adjusted HRs (95% CI) for CVD deaths was higher in smokers with and without metabolic syndrome (HR 3.19 [1.13 to 9.03] and 3.47 [1.48 to 8.12] in men; 4.94 [1.52 to 16.09] and 3.63 [1.75 to 7.50] in women, respectively). PAFs for CVD mortality in smokers with and without metabolic syndrome were 7.1% and 40.9% in men and 3.9% and 11.9% in women, respectively. The sum of PAF components due to smoking and/or metabolic syndrome was 60.4% in men and 17.0% in women.

**Table 4:** Hazard ratio and population attributable fraction for cardiovascular disease deaths according to the combination of smoking status and metabolic syndrome: NIPPON DATA90.

	Metabolic syndrome*	Number of participants	Person-years of follow-up	CVD deaths (n)	CVD mortality rate (per 1,000 person-years)	Adjusted hazard ratio( 95% CI) †			Estimated excess CVD deaths (n)	PAF component for CVD deaths (%)
Men										
Non smoker	-	480	6817	6	0.88	1.00				
	+	76	1109	1	0.90	1.32	(0.16	-10.97)	0.2	0.3
Past smoker	-	494	7036	18	2.56	2.13	(0.84	- 5.39)	9.5	11.0
	+	107	1494	3	2.01	1.49	(0.37	- 6.01)	1.0	1.1
Smoker	-	1343	18620	50	2.69	3.47	(1.48	- 8.12)	35.6	40.9
	+	252	3437	9	2.62	3.19	(1.13	- 9.03)	6.2	7.1
Women										
Non smoker	-	3,034	43585	38	0.87	1.00				
	+	397	5631	8	1.42	0.83	(0.38	- 1.78)	--	--
Past smoker	-	81	1042	1	0.96	1.06	(0.15	- 7.81)	0.05	0.1
	+	13	184	1	5.45	2.98	(0.41	-21.79)	0.6	1.1
Smoker	-	336	4627	10	2.16	3.63	(1.75	- 7.50)	7.2	11.9
	+	37	486	3	6.17	4.94	(1.52	-16.09)	2.4	3.9

*Metabolic syndrome were defined as follows: obesity (body mass index ≥ 25 kg/m^2^) plus any two of the following three factors: high blood pressure as blood pressure ≥ 130/85 mmHg or on treatment of hypertension, high blood glucose as blood glucose ≥ 110 mg/dl or on treatment of diabetes, dyslipidemia as triglyceride ≥ 150 mg/dl or high density lipoprotein cholesterol <40 mg/dl or on treatment of dyslipidemia.

†Hazard ratios were adjusted for age and drinking.

CVD, cardiovascular diseases; PAF, population attributable fraction; CI, confidence interval.

Table 5 shows adjusted HRs and PAF components due to the combination of smoking status and clustering of metabolic risk factors. Compared to non-smokers without metabolic risk factor clustering, adjusted HRs (95% CI) for CVD death in smokers with and without metabolic risk factor clustering were 5.85 (1.40 to 24.38) and 4.17 (0.98 to 17.71) for men, and 5.86 (2.41 to 14.23) and 4.56 (1.62 to 12.87) for women, respectively. PAF components for CVD mortality in non-smokers with metabolic risk factors clustering, smokers without metabolic risk factors clustering and smokers with metabolic risk factors clustering were 2.8%, 20.1% and 34.3% for men, and 18.7%, 6.4% and 10.9% for women, respectively.

**Table 5:** Hazard ratio and population attributable fraction for cardiovascular disease deaths according to the combination of smoking status and clustering of metabolic risk factors: NIPPON DATA90.

	Clustering of metabolic risk factors*	Number of participants	Person-years of follow-up	CVD deaths (n)	CVD mortality rate (per 1,000 person-years)	Adjusted hazard ratio (95% CI) †			Estimated excess CVD deaths (n)	PAF component for CVD deaths (%)
Men										
Non smoker	0 or 1	281	4002	2	0.50	1.00				
	2 ≤	275	3924	5	1.27	1.94	(0.38	-10.00)	2.4	2.8
Past smoker	0 or 1	282	4084	7	1.71	2.41	(0.50	-11.65)	4.1	4.7
	2 ≤	319	4446	14	3.15	3.37	(0.76	-14.96)	9.8	11.3
Smoker	0 or 1	819	11465	23	2.01	4.17	(0.98	-17.71)	17.5	20.1
	2 ≤	776	10592	36	3.40	5.85	(1.40	-24.38)	29.8	34.3
Women										
Non smoker	0 or 1	2,117	30554	14	0.46	1.00				
	2 ≤	1314	18661	32	1.71	1.55	(0.82	-2.95)	11.4	18.7
Past smoker	0 or 1	54	698	0	--			--	--	--
	2 ≤	40	527	2	3.79	3.08	(0.69	-13.81)	1.4	2.2
Smoker	0 or 1	222	3098	5	1.61	4.56	(1.62	-12.87)	3.9	6.4
	2 ≤	151	2016	8	3.97	5.86	(2.41	-14.23)	6.6	10.9

*Metabolic risk factors were any of the following four factors: obesity (body mass index ≥ 25 kg/m^2^), high blood pressure as blood pressure ≥ 130/85 mmHg or on treatment of hypertension, high blood glucose as blood glucose ≥ 110 mg/dl or on treatment of diabetes, dyslipidemia as triglyceride ≥ 150 mg/dl or high density lipoprotein cholesterol < 40 mg/dl or on treatment of dyslipidemia.

†Hazard ratios were adjusted for age and drinking.

CVD, cardiovascular diseases; PAF, population attributable fraction; CI, confidence interval.

## Discussion

The present report of a representative Japanese cohort showed that the majority of excess CVD deaths were observed in smokers without metabolic syndrome. The PAF component of CVD deaths in smokers without metabolic syndrome were 5 times higher than those in participants with metabolic syndrome in men (40.9% vs. 8.5%). The HR of CVD deaths in smokers without metabolic syndrome were also higher than non-smokers without metabolic syndrome, and it was similar to the HR in smokers with metabolic syndrome (3.47 vs. 3.19).

In Asian countries including Japan, there has been a rise in metabolic syndrome [7,25]. In these areas, prevalence of smoking has been higher than that in Western countries and smoking rates has been still increasing in younger women [14]. Previous studies have reported that obesity and smoking are risk factors for CVD [5,6,10,11]. The association of clustering of metabolic risk factors, including hyperglycemia, dyslipidemia, and hypertension, with CVD risk has also been widely reported [1,4,17,18]. Furthermore, a previous study from Japan reported that the effect of risk factor accumulation on CVD incidence was more evident among smokers than non smokers [17]. However, these previous reports did not show the attribution of the combination of smoking and metabolic syndrome (or obesity) to CVD events. To our knowledge, this is the first report showing that the majority of excess CVD deaths occurred in smokers without metabolic syndrome in a Asian population. A strength of our report is that the study was conducted in a 15-year cohort of nationwide representative Japanese samples.

In Japan, new health checkups and healthcare advice focusing on the metabolic syndrome to prevent CVD began in April 2008 through health insurance providers [7]. Our results support the necessity of intervention for people with metabolic syndrome because these people appear to be at higher CVD risk; however, PAF component in men and in women with metabolic syndrome were only 8.5% and 5.0%, respectively. On the other hand, the present study indicated that PAFs among smokers without metabolic syndrome were 40.9% in men and 11.9% in women; who are not the target population of the new health educational program in Japan. Moreover, not only PAF but also HR of smokers without metabolic syndrome was substantially higher. Thus the program might overlook a large population at an increased risk of CVD. Activities of smoking cessation for non-obese people would be still important for the prevention of CVD in Japan.

In the present study, we examined the association between each component of metabolic risk factors and CVD death. We conformed that current and past smoking, SBP and glucose were significant risk factors of CVD mortality in our study participants. Several previous reports revealed that clustering of metabolic risk factors increases CVD risk, irrespective of the presence of obesity [17,18]. When obesity was dealt with one of metabolic risk factors (not an essential factor) in the present study (Table 5), the PAF in smokers with metabolic risk factor clustering got larger in men (34.3%). However, even for smokers without metabolic risk factors clustering in the present study, PAF was 20.1% in men. This finding indicated that even if obesity is not essential for the diagnostic criteria of metabolic syndrome like the National Cholesterol Education Program (NCEP) [26], an intervention for smokers without clustering of metabolic risk factors would be also important for the prevention of CVD.

This study has several limitations. First, we used non-fasting blood samples and thus we might have misclassified several individuals with diabetes and dyslipidemia. Second, we used body mass index ≥ 25 kg/m^2 ^to define obesity and thus we might have misclassified individuals with abdominal obesity with higher waist circumference. However, this limitation would be ignorable because the correlation between BMI and waist circumstance is usually high enough. The cut-off point of body mass index for Japanese [21] is different from that for Asia-Pacific Region in the WHO definition (body mass index ≥ 23 kg/m^2^) [27], which may underestimate the PAF in Asian people. Third, we did not adjust for socioeconomic status in this study. However, all Japanese are covered by the national health insurance program and socioeconomic status would not limit access to treatment in Japan. Fourth, in this study, we used information on smoking habit from self-reported smoking history; this may cause recall and information biases. Fifth, the numbers of participants or CVD events were not enough to analyze according to the number of cigarettes per day; therefore, we did not consider the intensity of smoking in this paper.

## Conclusions

In conclusion, this long-term cohort study of representative Japanese samples indicated that CVD mortality in smokers without metabolic syndrome or without obesity was substantially high and a large proportion of excess deaths were observed in these groups. These findings suggest that intervention targeting on smokers, irrespective of the presence of metabolic syndrome (or obesity), is still important for the prevention of CVD death in relatively lean Japanese population with high smoking rate. This could apply to other Asian populations with high smoking rate but with lower prevalence of obesity compared with Western populations.

## Competing interests

The authors declare that they have no competing interests.

## Authors' contributions

All authors contributed to the concept, design, analysis, interpretation of data, and preparation of the manuscript. All authors read and approved the final manuscript.

## Pre-publication history

The pre-publication history for this paper can be accessed here:

http://www.biomedcentral.com/1471-2458/10/306/prepub
